# *Mendogiadiffusa* sp. nov. and an updated key to the species of *Mendogia* (Myriangiaceae, Dothideomycetes)

**DOI:** 10.3897/BDJ.9.e67705

**Published:** 2021-09-07

**Authors:** Vinodhini Thiyagaraja, Robert Lücking, Damien Ertz, Milan C. Samarakoon, Dhanushka N. Wanasinghe, Samantha C. Karunarathna, Ratchadawan Cheewangkoon, Kevin D. Hyde

**Affiliations:** 1 Department of Entomology and Plant Pathology, Faculty of Agriculture, Chiang Mai University, Chiang Mai 50200, Thailand Department of Entomology and Plant Pathology, Faculty of Agriculture, Chiang Mai University Chiang Mai 50200 Thailand; 2 Centre of Excellence in Fungal Research, Mae Fah Luang University, Chiang Rai 57100, Thailand Centre of Excellence in Fungal Research, Mae Fah Luang University Chiang Rai 57100 Thailand; 3 CAS Key Laboratory for Plant Biodiversity and Biogeography of East Asia (KLPB), Kunming Institute of Botany, Chinese Academy of Science, Kunming 650201, Yunnan, China CAS Key Laboratory for Plant Biodiversity and Biogeography of East Asia (KLPB), Kunming Institute of Botany, Chinese Academy of Science, Kunming 650201 Yunnan China; 4 World Agro forestry Centre East and Central Asia, Kunming 650201, Yunnan, China World Agro forestry Centre East and Central Asia, Kunming 650201 Yunnan China; 5 Botanischer Garten und Botanisches Museum, Freie Universität Berlin, Berlin, Germany Botanischer Garten und Botanisches Museum, Freie Universität Berlin Berlin Germany; 6 Research Department, Meise Botanic Garden, Nieuwelaan 38, BE-1860, Meise, Belgium Research Department, Meise Botanic Garden, Nieuwelaan 38, BE-1860 Meise Belgium; 7 Fédération Wallonie-Bruxelles, Service Général de l'Enseignement Supérieur et de la Recherche Scientifique, Rue A. Lavallée 1, BE-1080, Bruxelles, Belgium Fédération Wallonie-Bruxelles, Service Général de l'Enseignement Supérieur et de la Recherche Scientifique, Rue A. Lavallée 1, BE-1080 Bruxelles Belgium; 8 Innovative Agriculture Research Centre, Faculty of Agriculture, Chiang Mai University, Chiang Mai 50200, Thailand Innovative Agriculture Research Centre, Faculty of Agriculture, Chiang Mai University Chiang Mai 50200 Thailand; 9 Department of Biology, Faculty of Science, Chiang Mai University, Chiang Mai 50200, Thailand Department of Biology, Faculty of Science, Chiang Mai University Chiang Mai 50200 Thailand; 10 Research Center of Microbial Diversity and Sustainable Utilization, Chiang Mai University, Chiang Mai 50200, Thailand Research Center of Microbial Diversity and Sustainable Utilization, Chiang Mai University Chiang Mai 50200 Thailand

**Keywords:** one new species, morphology, multilocus phylogeny, saprotroph, taxonomy

## Abstract

**Background:**

*Mendogia* belongs to Dothideomycetes and its members are epiphytic on living bamboo culms or palms and distributed in tropical regions. Currently, the genus comprises seven species. Another collection resembling *Mendogia* was collected from the leaves of *Fagales* sp. in Thailand. Morphological characteristics and multilocus phylogenetic analyses, using ITS, LSU and SSU sequences, showed that the fungus is new to science, described herein as *Mendogiadiffusa*. *Mendogiadiffusa* is characterised by apothecial ascostromata, a carbonised epithecium, dark brown setae on the ascostromatal surface, hyaline paraphysoids, ovoid to clavate asci and oblong to elliptical, muriform ascospores. The fungus has a dark pigmented surface and is occasionally facultatively associated with patches of green algae, but not actually lichenised. Instead, the fungus penetrates the upper leaf surface, forming dark pigmented isodiametric cells below the epidermis.

**New information:**

Re-examination of specimens of *M.chiangraiensis*, *M.macrostroma* and *M.yunnanensis* revealed the absence of algal associations. The status of *Mendogiaphilippinensis* (= *M.calami*) and *M.bambusina* (= *Uleopeltisbambusina*) was established, based on morphological comparisons and previous studies. Comprehensive morphological descriptions with phylogenetic analyses support *M.diffusa* as a novel species in *Myriangiaceae*. An updated key to the known species of the genus is also provided.

## Introduction

Dothideomycetes is the largest class in Ascomycota, comprising 19,000 species, including saprotrophs, pathogens, endophytes, epiphytes, fungicolous, lichenised and lichenicolous taxa (Hyde et al. 2013, Hongsanan et al. 2020). *Myriangiales* was introduced by Starbäck (1899), based on species producing crustose ascostromata and muriform ascospores in the Dothideomycetes (Hyde et al. 2013). These species occur as pathogens, saprobes or epiphytes on bark, leaves and branches of plants (Dissanayake et al. 2014, Jayawardena et al. 2014), while some are rock-inhabiting (Ruibal et al. 2009). Kirk et al. (2008) included *Cookellaceae*, *Elsinoaceae* and *Myriangiaceae* in *Myriangiales*. Based on molecular phylogenetic studies, Lumbsch and Huhndorf (2010) accepted only *Elsinoaceae* and *Myriangiaceae* within *Myriangiales*, whereas *Cookellaceae* was treated as Dothideomycetes *incertae sedis*. This classification was accepted in subsequent studies (Hyde et al. 2013, Dissanayake et al. 2014, Jayawardena et al. 2014, Wijayawardene et al. 2014 Dai et al. 2017, Wijayawardene et al. 2017, Wijayawardene et al. 2018, Hongsanan et al. 2020, Jiang et al. 2020, Wijayawardene et al. 2020). *Myriangiaceae* is a poorly known family (Dissanayake et al. 2014) and comprises 11 genera. These are *Anhellia*, *Ascostratum*, *Butleria*, *Dictyocyclus*, *Eurytheca*, *Hemimyriangium*, *Mendogia*, *Micularia*, *Myriangium*, *Uleomyces* and *Zukaliopsis* (Hongsanan et al. 2020, Wijayawardene et al. 2020). Members in *Myriangiaceae* occur mainly in tropical and sub-tropical areas (Boedijn 1961, Barr 1979).

*Mendogia* was introduced by Raciborski (1900), based on the single species *M.bambusina* collected on bamboo in Indonesia. This genus was, for some time, placed in *Schizothyriaceae* (von Arx and Müller 1975). However, Dai et al. (2017) provided the first molecular data for *M.macrostroma* and transferred this genus to *Myriangiaceae*, based on morphological and phylogenetic analyses. Seven species are currently recognised within this genus (Jiang et al. 2020). They are characterised by small to large, black, flattened, solitary to scattered, superficial ascostromata with a centrally raised area, subglobose to clavate, bitunicate, (6–)8(–10)-spored asci with a distinct ocular chamber and elliptical, muriform, hyaline ascospores (Jiang et al. 2020). The species of *Mendogia*, thus far known, are exclusively epiphytic on living bamboo culms or palms and are found in Brazil, China, Indonesia, Philippines and Thailand (Raciborski 1900, Hennings 1904, von Arx and Müller 1975, Dai et al. 2017). *Mendogia* is distinguished from other genera of this family by its larger ascostromata, thick peridium, carbonaceous outer cells, pseudoparenchymatous inner cells and muriform ascospores (Phookamsak et al. 2016).

This study introduces a new species of *Mendogia* that appeared unusual due to its growth on leaves and its occasional, facultative association with patches of green algae. We conducted a detailed investigation to resolve the identity of our newly-collected material, including morphological and chemical assessments. The phylogenetic position of the taxon was investigated, based on Maximum Likelihood and Bayesian analyses of combined ITS, LSU and SSU sequences. We further re-examined herbarium collections of *Mendogiachiangraiensis*, *M.macrostroma* and *M.yunnanensis* to test potential associations with algae. Additionally, morphological comparisons between closely-related taxa have led to reclassify several species in *Mendogia* (*M.philippinensis* (= *M.calami*) and *M.bambusina* (= *Uleopeltisbambusina*)). We, therefore, provided an updated key to the genus.

## Materials and methods

### Morphological analysis

The fungal material was collected in Phayao, Thailand. Herbarium specimens of *Mendogiachiangraiensis*, *M.macrostroma* and *M.yunnanensis* were loaned from Mae Fah Luang University Herbarium (MFLU), Chiang Rai, Thailand. Fungal structures on the substrate were observed with a stereomicroscope and micro-morphological features were examined and photographed using a Nikon Eclipse E600 fluorescence microscope with a Canon 750D digital camera. Hand sections of the ascomata were mounted in water, 5% potassium hydroxide (KOH), 5% Lugol's solution and Trypan blue. All microscopic measurements were measured in water and images were made with Tarosoft Image Frame Work (0.9.0.7) and processed with Adobe Photoshop CS6 Extended 10.0 software (Adobe Systems, San Jose, CA, USA). The newly-proposed synonymies were established, based on revision of available data from previous studies. The holotype specimen of *M.diffusa* was deposited in the Mae Fah Luang University (MFLU) Herbarium, Chiang Rai, Thailand.

### DNA extraction, PCR amplification and sequencing

The E.Z.N.A. Forensic DAT (D3591 – 01, Omega Bio–Tek, Guangzhou, China) kit was used to extract DNA, following the manufacturer’s instructions. DNA samples that were intended for use as a template for PCR were stored at 4°C for use in regular work; long-term storage was at -20°C. The small and large subunits (SSU, LSU) of the nuclear ribosomal RNA gene, as well as the internal transcribed spacer (ITS) region were amplified with primer pairs NS1/NS4 (White et al. 1990), LR0R/LR5 (Vilgalys and Hester 1990, Hopple 1994) and ITS5/ITS4 (White et al. 1990), respectively. PCR amplification was performed using a final volume of 25 µl, comprised of 2.0 µl of DNA template, 1 µl of each forward and reverse primer, 12.5 µl of Taq PCR Super Mix and 8.5 µl of sterilised water. Cycling conditions were as follows: initial denaturation at 94°C for 3 min; followed by 40 cycles of denaturation at 94°C for 30 s, annealing at 55°C for 50 s, elongation at 72°C for 1 min; and a final extension at 72°C for 10 min. PCR products were examined on 1% agarose electrophoresis gels and stained with ethidium bromide. Purification and DNA sequencing were performed at Shanghai Sangon Biological Engineering Technology and Services Co. (Shanghai, P.R. China). Forward and reverse sequence reads were assembled and manually edited in Bioedit. Generated sequences were submitted to NCBI GenBank (https://www.ncbi.nlm.nih.gov/genbank/). Alignments and phylogenetic trees were submitted to TreeBASE with Submission ID: 28050.

### Phylogenetic analyses and species recognition

The newly-generated sequences were BLAST-searched against the NCBI GenBank standard nr/nt database (https://blast.ncbi.nlm.nih.gov/BLAST.cgi). Sequences of closely-related taxa for *Myriangiales* were downloaded from GenBank. We failed to generate sequences for the translation elongation factor 1-alpha (TEF1) using the primer pair EF1-983F/EF1-2218R with the PCR conditions recommended in Jiang et al. (2020). As a result, our phylogenetic analyses were carried out using ITS, LSU and SU sequences (Table 1). *Columnosphaeriafagi* (CBS 171.93), *Dothideainsculpta* (CBS 189.58), *D.sambuci* (DAOM 231303), *Dothioracannabinae* (CBS 737.71) and *Sydowiapolyspora* (CBS 116.290) were used as outgroup taxa (Jiang et al. 2020).

Phylogenetic analyses of both individual and combined aligned data were performed under Maximum Likelihood (ML) and Bayesian Inference (BI) criteria. Multiple alignments were automatically performed for each locus with MAFFT v. 7 (http://mafft.cbrc.jp/alignment/server/index.html, Katoh et al. 2017). Terminal ends of sequences and ambiguous regions were trimmed manually using BioEdit v.7.0.5.2 (Hall 2001) and excluded from the analysis. The phylogenetic web tool “ALTER” (Glez-Peña et al. 2010) was used to convert sequence alignment from FASTA to NEXUS format for Bayesian analysis. The estimated model of ML and Bayesian analyses were performed independently for each locus using MrModeltest v.2.2 (Nylander 2008). ML analysis was perfomed in IQ-TREE web server under different partitions (Nguyen et al. 2015) for SSU, LSU, ITS1, 5.8S and ITS2 gene regions, with default parameters. MrBayes v. 3.1.2 was used to perform Bayesian analysis (Huelsenbeck and Ronquist 2001). Markov Chain Monte Carlo sampling (MCMC) was run for 5,000,000 generations and the trees were sampled every 100^th^ generation. The first 10% of trees that represented the burn-in phase were discarded and only the remaining 90% of trees were used for calculating posterior probabilities (PP) for the majority rule consensus tree. The resulting trees were visualised in FigTree v.1.4.0 (http://tree.bio.ed.ac.uk/software/figtree/), then edited in Microsoft PowerPoint 2013 and converted to a jpeg file using Adobe Photoshop CS6 (Adobe Systems, USA).

## Taxon treatments

### Mendogia diffusa

Thiyagaraja, Ertz, Lücking, Samarak. and K.D. Hyde sp. nov.

AE26DD54-AD2A-51B3-B587-CE68C37D8AD8

IF 558292

FoF 09466

#### Materials

- **Type status:**Holotype. **Occurrence:** recordedBy: Milan C. Samarakoon; occurrenceID: MFLU 20-0541; **Taxon:** kingdom: Fungi; phylum: Ascomycota; class: Dothideomycetes; order: Myriangiales; family: Myriangiaceae; genus: Mendogia; specificEpithet: *diffusa*; scientificNameAuthorship: Thiyagaraja, Ertz, Lücking, Samarak. and K.D. Hyde; **Location:** continent: Asia; country: Thailand; stateProvince: Phayao; locality: Phu Sang; **Identification:** identificationID: MFLU 20-0541; identifiedBy: Vinodhini Thiyagaraja; dateIdentified: 4 Dec 2018; **Record Level:** modified: 4 December 2018; institutionID: MFLU; institutionCode: Mae Fah Luang University

#### Description

**Figure 1.**
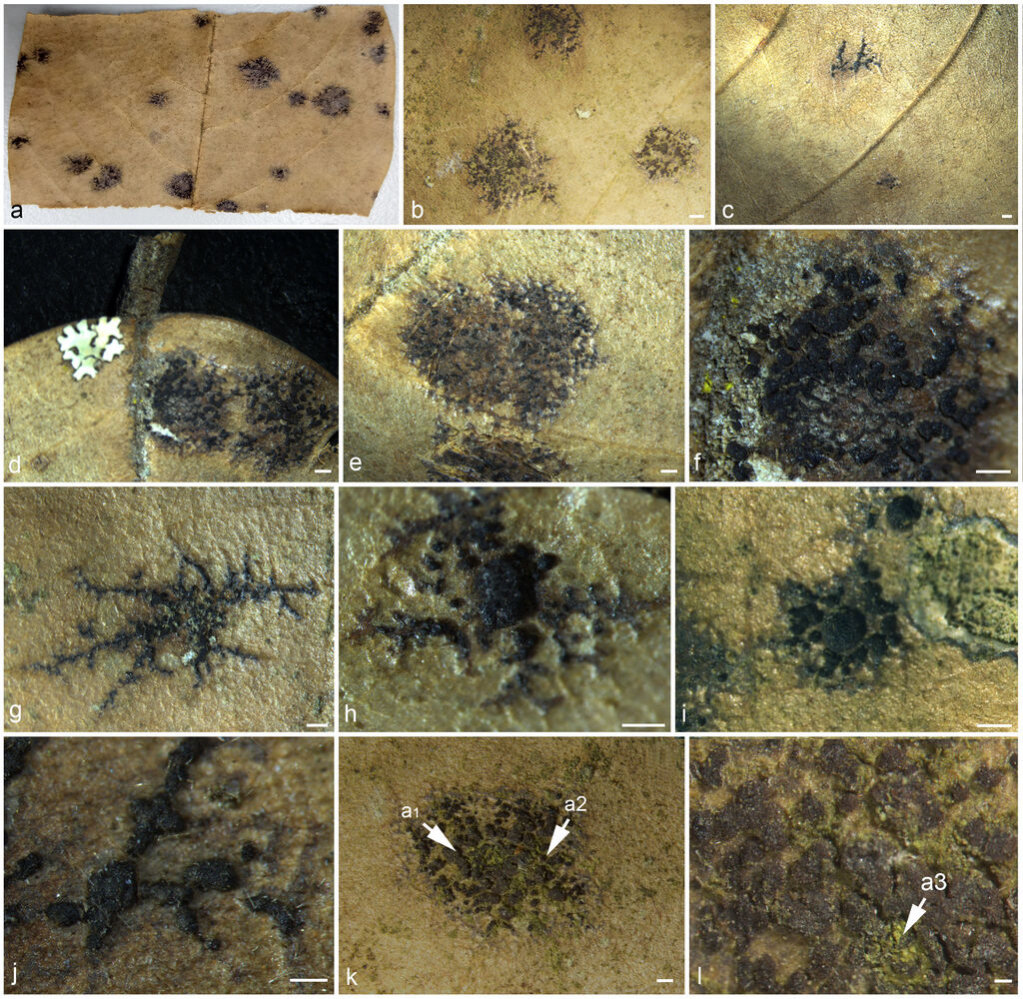
*Mendogiadiffusa* (MFLU 20-0541) **a, b, d–l.** Ascomata on upper leaf surface; **c.** Ascomata on lower leaf surface; arrows point the algae. Scale bars: b = 1000 µm, g–j = 500 µm

**Figure 2.**
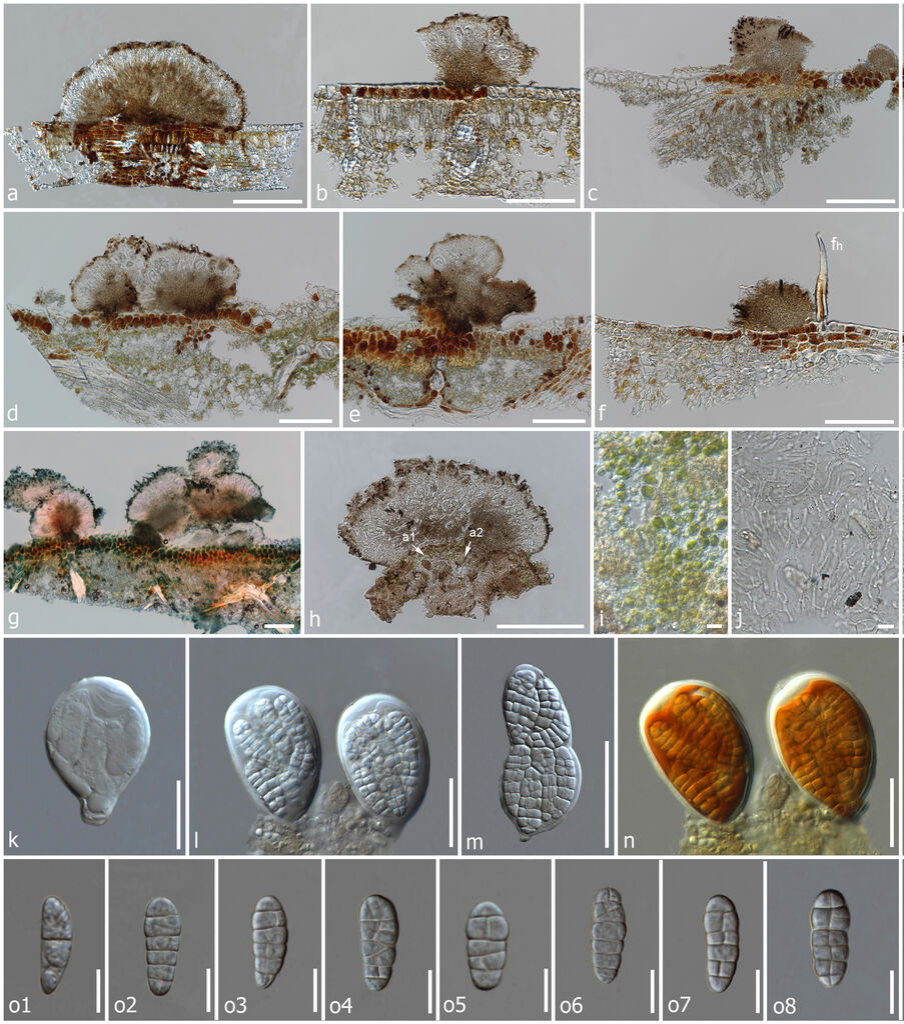
*Mendogiadiffusa* (MFLU 20-0541, holotype) **a–e.** Vertical sections of ascomata in water (upper surface); **f.** Vertical section of an ascoma in water (lower surface); fh, hair-like structure on leaf; **g.** Ascomata in trypan blue; **h, i.** (a1, a2) Algae; **j.** Paraphysoids in water; **k–m.** Asci in water; **n.** Asci in 5% KOH stained with Lugol's solution; **o1–o8.** Ascospores in water. Scale bars: (a–g) = 200 µm, (h–j) = 5 µm, (k–n) = 30 µm, (g–j) = 30 µm, (o1–o8) = 10 µm

*Saprotrophic* on dead leaves. *Thallus* absent (Fig. 1 and Fig. 2). **Sexual morph**: *Ascomata* scattered in dense, pseudostromatic, irregularly stellate groups over an effuse, thallus-like, dark structure, with thin covering layer, superficial, solitary or gregarious, easily removed from the host surface, carbonaceous, ovoid to sub-globose, black, abundant, with numerous external dark brown setae on the epithecium, which are branched at the end, individual loci (120-)225–410 µm wide, 250–180 µm high. *Epithecium* 16–33 µm thick, distinct, dark brown. *Hymenium* 40–95 µm high, hyaline. *Hypothecium* 35–75 µm thick, distinct, thicker in the centre, brownish, infrequently with free-living unicellular algae below the hypothecium. *Excipulum* inconspicuous. *Paraphysoids* 1.1–3.3 µm thick, abundant, anastomosing, branched, not or slightly enlarged at the apex. *Asci* 45–70 × 25–35 µm (x¯ = 57.5 × 30 µm, n = 20), 8-spored, bitunicate, fissitunicate, ovoid to clavate, tholus thickened, tip blunted, with poorly developed stipe, ascus wall apically thickened with well-developed ocular chamber, concave. *Ascospores* 15–25 × 6–10 µm (x¯ = 20 × 8 µm, n = 20), irregularly arranged, hyaline, oblong to elliptical, both ends bluntly tapered, muriform, with 5–6 transverse septa, 3–6 longitudinal septa, slightly constricted at each septum, smooth-walled, without gelatinous sheath, occasionally asymmetrical. *Hymenium* I–, KI–, *Asci* I–, KI–. **Asexual morph**: Undetermined.

#### Etymology

Referring to the morphology of the fungus with ascostromata that are diffuse and spread extensively on the leaves.

#### Distribution

##### Habitats and Distribution

On dead leaves of *Fagales* sp. Thus far, only known from Thailand, Phayao Province, Phu Sang District.

#### Notes

*Mendogiadiffusa* is the first reported species in the genus from dead dicotyledonous leaves. Other species were mostly reported from bamboo culms, with the exception of *M.manaosensis* that is reported from palm leaves (Vitória 2012, Dai et al. 2017) and *M.philippinensis* (= *M.calami*) that is found on living leaves of *Calamus* palms (Jiang et al. 2020). In those species, ascostromata do not penetrate the leaf surface and they also differ from *M.diffusa* in the sharply delimited ascostromata; and *M.philippinensis* further differs in the smaller ascospores. The new taxon shares morphological characteristics with *Mendogiabambusina*: carbonaceous peridium, paraphysoid-like filaments, similar asci and ascopores. However, *M.diffusa* differs in the absence of ascostromata, presence of setae (Dai et al. 2017), the type of habitat (*Fagales* leaves vs. bamboo or palms culms) and its distribution (Thailand vs. Indonesia) (Hyde et al. 2013, Dai et al. 2017).

### Mendogia philippinensis

(Syd. & P. Syd.) Arx & E. Müll., Stud. Mycol. 9: 29 (1975).

0A2F1579-95DB-51F3-B4EE-F0295FB001ED

Mendogia philippinensis *Pleiostomella philippinensis
*
Mendogia philippinensis *Mendogiacalami*; Basionym: *Pleiostomellaphilippinensis* Syd. & P. Syd., Annls mycol. 15(3/4): 221 (1917); Type: The Philippines, Biliran, 1914, RC McGregor 18371 (S-F61491).; Syn. nov.: *Mendogiacalami* H.B. Jiang, Phookamsak and K.D. Hyde, in Jiang, Phookamsak, Xu, Karunarathna, Mortimer and Hyde, Mycol. Progr. 19: 47 (2020); Type: The Philippines, Mt. Makiling, S. A. Reyes 3367a, (S-F48343).

#### Notes

*Mendogiacalami* was recently introduced from leaves of *Calamus* sp. in the Philippines (Jiang et al. 2020). However, there are no discernible differences between *M.philippinensis* and *M.calami*, neither in phenotype nor in substrate ecology. Jiang et al. (2020) did not discuss *M.philippinensis* when establishing *M.calami* and the difference implied in the table and key (ascostroma size, number of longitudinal septa) are either due to age (ascostroma size) or they are non-existent (the ascospores of *M.calami* have mostly one, rarely two longitudinal septa in the photographs and the protologue of *M.philippinensis* also indicates mostly one longitudinal septum) (Sydow and Sydow 1917). This synonymy needs further testing with molecular data as previous studies on palms have shown that the taxa on different palm species differ (Konta et al. 2016, Konta et al. 2017) as they may have derived from endophytes.

### Mendogia bambusina

Racib., Parasit. Alg. Pilze Java's (Jakarta) 3: 31 (1900)

BBEB7833-661E-57B0-98E9-3432A165538D

Mendogia bambusina *Uleopeltis bambusina
*
Mendogia bambusina *Ital. 1 (Fasc. 3): 159 (1862).*; Syn. nov.: *Uleopeltisbambusina* Syd. & P. Syd., Annls mycol. 12(6): 565 (1914); *Ital. 1 (Fasc. 3): 159 (1862).* Type: The Philippines, Luzon, Bulacan Prov., Angat, 1913, M Ramos, Bur. Sci. 21852 (GZU, S-F5988).

#### Notes

*Uleopeltis* was introduced to accommodate *U.manaosensis* and later the second species *U.bambusina* added to this genus (Hennings 1904, Dai et al. 2017). *Uleopeltismanaosensis* was synonymised under *Mendogia*, while *U.bambusina* remained in *Uleopeltis* which was collected from bamboo culms in he Philippines (Hennings 1904, von Arx and Müller 1975, Dai et al. 2017). The species lacks molecular data and shares similar morphological characteristics with the type species of *Mendogia* (von Arx and Müller 1975). Dai et al. (2017) gave spores of the type material of *Mendogiabambusina* as 13.5–25 × 5–8 µm, but mature ascospores in the photographs are 15–21 × 7–9 µm. Raciborski (1900) gave the ascospores as 17–19 × 8 µm for *M.bambusina*. This supports the assessment of von Arx and Müller (1975) that *M.bambusina* and *Uleopeltisbambusina* are conspecific. The synonymisation is formalised here. The report of *M.bambusina* from Brazil on palm leaves (Vitória 2012) has been documented with morphological and anatomical photographs and agrees well with the material from the Paleotropics. The African *Pleiostomellahalleriae* (Doidge 1921) will also key out close to *M.bambusina* and may represent another synonym. It is the only other species described in *Pleiostomella*, a synonym of *Mendogia*, but has apparently never been dispositioned. Unfortunately, no type was indicated and a total of six collections on two host species (leaves of *Halleriaelliptica* and *H.lucida*) were listed. The ascus and ascospore dimensions (50–70 × 20–33 µm; 22–24 × 9–10 µm) partly fit *M.bambusina*, but Doidge described two types of asci, one ovate and ca. 50 × 30 µm and the other clavate and ca. 65–70 × 20–25 µm. The latter fits *M.bambusina*, whereas the former does not conform to any of the species recognised here. Revision of all paratypes is necessary to assess the taxonomic status of this material (Sydow and Sydow 1917).

## Identification Keys

### Key to the species of *Mendogia*

**Table:** 

1	Ascomata scattered in dense, pseudostromatic, irregularly stellate groups over an effuse, thallus-like, dark structure, with thin covering layer, interascal hyphae forming distinct paraphysoids, asci 45–70 × 25–35 µm, ascospores 15–25 × 6–10 µm, on dead dicotyledonean leaves, Thailand	* Mendogia diffusa *
–	Ascomata one to many immersed in sharply delimited, rounded ascostromata, without associated thallus-like structure, interascal hyphae, asci and ascospores variable, on living bamboo culms or palm leaves	2
2	Ascospores narrowly oblong, transversely septate, 30–55 × 3.5–4.5 µm, interascal hyphae forming sparsely branched paraphysoids, asci cylindrical-clavate, 85–120 × 10–12 µm, Brazil	*Mendogiamanaosensis* (= *Uleopeltismanaosensis*)
–	Ascospores broadly oblong to somewhat tapering, muriform, interascal hyphae variable, asci broadly oblong to obclavate	3
3	Ascostromata with distinct chambers appearing peritheciiform in cross section, but forming dense, concentric structures, with the asci in a single layer formed at the bottom of the chambers (type II), interascal hyphae forming more or less distinct paraphysoids, asci 45–55 × 16–20 µm, ascospores 14–18 × 5–6.5 µm, on living palm leaves, Philippines	*Mendogiaphilippinensis* (= *Pleiostomellaphilippinensis*) (= *Mendogiacalami*)
–	Ascostromata indistinctly chambered (arthothelioid) or asci in concentric structures mostly towards the periphery, with the asci irregularly dispersed in irregular layers (type I), on bamboo culms (rarely on palm leaves) interascal hyphae forming indistinct paraphysoids or textura angulate	4
4	Interascal hyphae forming indistinct paraphysoids, asci developing in concentric structures mostly towards the periphery, 17–25 µm broad, ascospores 15–28 × 7–11 µm, without gelatinous caps, on bamboo culms or palm leaves, USA, Brazil, Indonesia, Philippines	*Mendogiabambusina* (= *Uleopeltisbambusina*)
–	Interascal hyphae forming a textura angulata, asci and ascospores variable	5
5	Ascostromata 5–20 mm diam., asci 70–85 × 28–35 µm, ascospores 20–27 × 9–11 µm, without gelatinous sheath or caps, on bamboo culms, Thailand	* Mendogia macrostroma *
–	Ascostromata 1–5 mm diam., asci and ascospores variable in size, but ascospores with thin gelatinous sheath and distinct gelatinous caps	6
6	Asci 55–75 × 25–30 µm, ascospores 19–23 × 8–11 µm, on bamboo culms, China	* Mendogia yunnanensis *
–	Asci 75–165 × 30–40 µm, ascospores 25–35 × 12–16 µm, on bamboo culms, Thailand	* Mendogia chiangraiensis *

## Analysis

### Phylogenetic analyses

The genera of *Myriangiaceae* were well recovered, as studied in Jiang et al. (2020). The final alignment comprised 50 strains including the new strain and 2469 nucleotide positions. The topologies of the single gene markers tree and the tree topology obtained from the combined five-locus (SSU, LSU, ITS1, 5.8S, ITS2) dataset were congruent. Our phylogenetic analyses supported the placement of *Mendogiadiffusa* within *Mendogia*. The average standard deviation of split frequencies at the end of total MCMC generations was calculated as 0.0024 in the Bayesian analysis.

## Discussion

**Figure 3.**
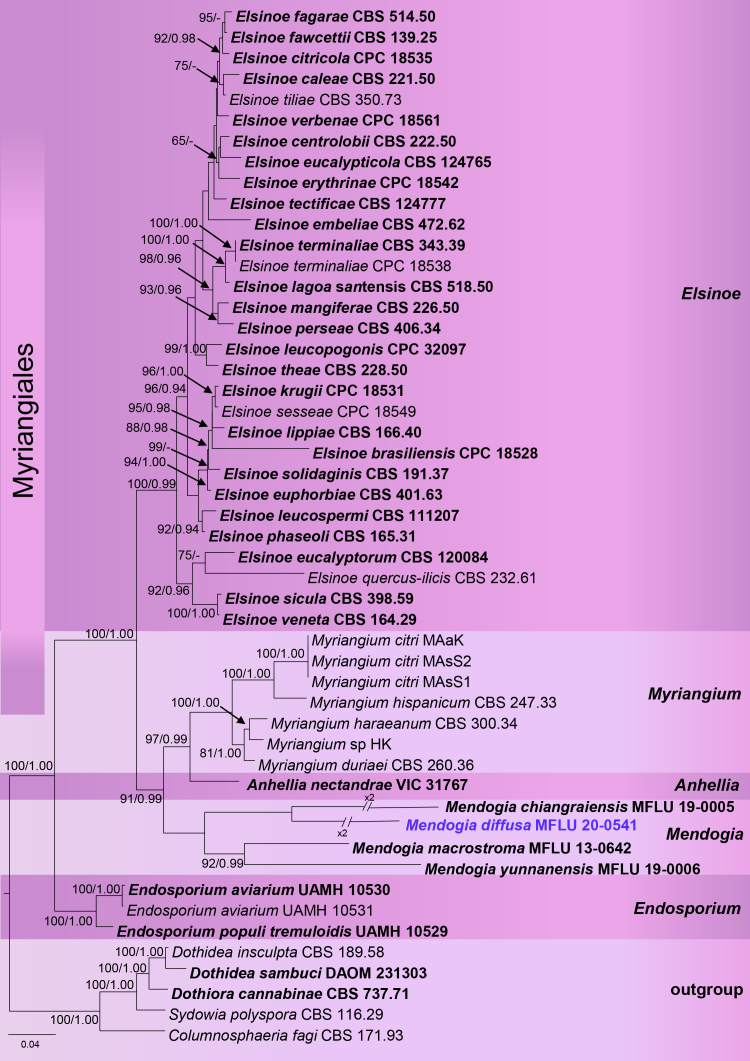
Phylogeny of Myrangiales reconstructed from a multilocus dataset with SSU, LSU, ITS1, 5.8S and ITS2. The topology is the result of ML inference performed with IQ-TREE. ML bootstrap support values = 65% and Bayesian posterior probabilities = 0.95 are presented above each branch. Ex-type strains are shown in black bold; the new species is highlighted in blue bold font.

*Mendogia* has previously been recorded from monocotyledons, but, in the present case, was collected on a dicotyledon, indicating many more species are likely to be discovered. Other species currently recognised in *Mendogia* (see key above) differ from the new species in the sharply delimited ascostroma (Dai et al. 2017, Jiang et al. 2020), which renders the diffusely delimited ascomata (Fig. 1) as the most diagnostic feature of *M.diffusa*. In terms of ascospore size, *M.bambusina*, *M.macrostroma* and *M.yunnanensis* are closely related to *M.diffusa*. Apart from the sharply delimited ascostromata and the usually bambusicolous habit of all three species, *M.bambusina* has narrower asci and *M.macrostroma* differs in the much larger ascostromata (Raciborski 1900, Dai et al. 2017, Jiang et al. 2020). The internal anatomy of the ascomata of *M.diffusa* is also distinctive, with easily discernible paraphysoids (Fig. 2). *Mendogiamanaosensis* and *M.philippinensis* (= *M.calami*) also form paraphysoid-like interascal hyphae, whereas in *M.bambusina*, these are less distinctive and, in *M.chiangraiensis*, *M.macrostroma* and *M.yunnanensis*, the interascal hyphae form a *textura angularis* (Raciborski 1900, Hennings 1904, Sydow and Sydow 1917, von Arx and Müller 1975, Dai et al. 2017, Jiang et al. 2020). This variation in morphology and internal anatomy of such closely-related species is remarkable, especially given that, in our phylogenetic analysis, *M.diffusa* and *M.chiangraiensis* formed a sister clade to *M.macrostroma* and *M.yunnanensis* (Fig. 3), although without support. The new taxon shows more than 2% nucleotide differences in the ITS region compared to other *Mendogia* species. This, along with the discussed morphological differences, supports recognition as a new species (Jeewon and Hyde 2016). Unfortunately, DNA sequences are lacking for three of the seven recognised species in the genus: *M.bambusina*, *M.manaosensis* and *M.philippinensis* (= *M.calami*). *Mendogiadiffusa* should not be confused with the superficially similar *Diplothecatunae* in the same family (Dissanayake et al. 2014). The latter also forms ascomata scattered in dense groups instead of sharply delimited ascostromata, but differs in the broad, globose asci and the much thicker covering layer of the ascomata.

*Mendogiadiffusa* was found on dead leaves and the fungal structures penetrate the upper epidermis of the leaf surface, turning the epidermal cells into a dark pigmented layer (Fig. 2). Such dark pigmented cells are absent where the ascomata are not observed. Some ascostromata observed were found to loosely associate with algal colonies (Fig. 1). The algae are probably trentepohlioid, 3–5 µm thick, rounded to slightly elongate and greenish. However, since these are absent from most of the ascostromata and no closer anatomical associations or penetration structures were detected, we assume that this association is opportunistic, the algae is taking advantage of the microrelief formed by the ascostromata to colonise the otherwise smooth leaf surface. While the ascostromata were detected on dead leaves, it is unclear whether the fungus is also present on living leaves and how common is the observed opportunistic association with algae. It is possible that *M.diffusa* indirectly benefits from the presence of the algae as an additional carbon source, through leaching or by decomposing dead algal cells. Similar cases of loose associations have been reported from saxicolous biocoenoses where rock-inhabiting fungi are often growing together with algae or cyanobacteria (Muggia et al. 2013). Muggia et al. (2016) found alpine rock lichens to be associated with members of *Myriangiales*.

## Supplementary Material

XML Treatment for Mendogia diffusa

XML Treatment for Mendogia philippinensis

XML Treatment for Mendogia bambusina

## Figures and Tables

**Table 1.** Taxa used in this study for the phylogenetic analyses of combined SSU, ITS and LSU sequence data and their GenBank accession numbers. The newly-generated sequences are given in black boldface.

		GenBank Accessions Number		
Species	Strain	ITS	LSU	SSU
* Anhellia nectandrae *	VIC 31767	NR_111700	NG_042604	-
* Columnosphaeria fagi *	CBS 171.93	KT693737	AY016359	AY016342
* Dothidea insculpta *	CBS 189.58	AF027764	NG_027643	DQ247810
* Dothidea sambuci *	DAOM 231303	NR_111220	NG_027611	NG_012432
* Dothiora cannabinae *	CBS 737.71	NR_144904	DQ470984	NG_062696
* Elsinoe brasiliensis *	CPC 18528	NR_148130	JN940394	NG_064989
* Elsinoe caleae *	CBS 221.50	NR_148131	NG_064001	-
* Elsinoe centrolobii *	CBS 222.50	NR_148132	KX886969	NG_062717
* Elsinoe citricola *	CPC 18535	NR_148133	KX886970	JN940559
* Elsinoe embeliae *	CBS 472.62	NR_148136	KX886974	-
* Elsinoe erythrinae *	CPC 18542	KX887214	KX886977	JN940550
* Elsinoe eucalypticola *	CBS 124765	NR_132834	KX886978	-
* Elsinoe eucalyptorum *	CBS 120084	NR_155080	KX886979	-
* Elsinoe euphorbiae *	CBS 401.63	NR_148137	KX886980	-
* Elsinoe fagarae *	CBS 514.50	NR_148138	KX886981	-
* Elsinoe fawcettii *	CBS 139.25	NR_148139	KX886982	-
* Elsinoe krugii *	CPC 18531	NR_148150	KX886998	NG_064987
* Elsinoe lagoa-santensis *	CBS 518.50	NR_148151	KX887002	-
* Elsinoe leucopogonis *	CPC 32097	NR_159836	NG_064551	-
* Elsinoe leucospermi *	CBS 111207	NR_148154	KX887005	-
* Elsinoe lippiae *	CBS 166.40	NR_148155	NG_063985	-
* Elsinoe mangiferae *	CBS 226.50	NR_148156	KX887012	-
* Elsinoe perseae *	CBS 406.34	NR_148160	NG_063977	-
* Elsinoe phaseoli *	CBS 165.31	NR_148161	KX887026	NG_062718
* Elsinoe quercus-ilicis *	CBS 232.61	NR_148164	-	-
* Elsinoe sesseae *	CPC 18549	KX887288	KX887051	JN940561
* Elsinoe sicula *	CBS 398.59	NR_148170	KX887052	-
* Elsinoe solidaginis *	CBS 191.37	NR_148171	KX887053	-
* Elsinoe tectificae *	CBS 124777	NR_148172	KX887055	-
* Elsinoe terminaliae *	CBS 343.39	NR_148173	KX887056	-
* Elsinoe terminaliae *	CPC 18538	JN943497	JN940371	JN940560
* Elsinoe theae *	CBS 228.50	NR_148174	KX887058	-
* Elsinoe tiliae *	CBS 350.73	KX887296	KX887059	-
* Elsinoe veneta *	CBS 164.29	NR_148175	NG_059194	NG_062714
* Elsinoe verbenae *	CPC 18561	NR_148176	NG_059208	NG_064988
* Endosporium aviarium *	UAMH 10530	NR_111286	NG_059195	NG_016524
* Endosporium aviarium *	UAMH 10531	EU304352	EU304353	-
* Endosporium populi-tremuloidis *	UAMH 10529	EU304347	EU304348	EU304346
* Mendogia diffusa *	**MFLU 20-0541**	**MW854639**	**MW854637**	**MW854638**
* Mendogia chiangraiensis *	MFLU 19-0005	MK433591	-	MK433594
* Mendogia macrostroma *	MFLU 13-0642	NR_154192	KU863104	NG_065082
* Mendogia yunnanensis *	MFLU 19-0006	-	MK433593	MK433601
* Myriangium citri *	MAaK	KU720544	KU720541	-
* Myriangium citri *	MAsS1	KU720543	KU720539	-
* Myriangium citri *	MAsS2	KU720542	KU720540	-
* Myriangium duriaei *	CBS 260.36	MH855793	NG_027579	AY016347
* Myriangium hispanicum *	CBS 300.34	MH855532	MH867034	-
* Myriangium haraeanum *	CBS 247.33	MH855426	KX887067	-
*Myriangium* sp.	HK	KR909171	-	-
* Sydowia polyspora *	CBS 116.29	MH 855019	DQ678058	DQ678005
